# Introduction to the Greater Bay Area (Huangpu) International Algorithm Case Competition

**DOI:** 10.1093/nsr/nwad154

**Published:** 2023-05-25

**Authors:** Hai Zhang, Xing Xu

**Affiliations:** Northwest University and Pazhou Laboratory (Huangpu), China; University of Electronic Science and Technology of China and Pazhou Laboratory (Huangpu), China; Northwest University and Pazhou Laboratory (Huangpu), China; University of Electronic Science and Technology of China and Pazhou Laboratory (Huangpu), China

## COMPETITION OVERVIEW

The regional government of Huangpu district in the city of Guangzhou invited Pazhou Laboratory (PZL) in July of 2022 to host an international contest of algorithms of artificial intelligence (AI) and big data. The contest was named the Guangdong-Hong Kong-Macau Greater Bay Area (Huangpu) International Algorithm Case Competition. The competition provided PZL, as a national research facility, with an opportunity to lead the development of AI and big data algorithms in the Greater Bay Area. The goal was to create the first international algorithm competition in the area as an avenue to promote innovation and elevate the digital economy of the region.

PZL conducted comprehensive surveys of major national and international competitions in order to maximize the attendance and impact of the event. They invited top experts of AI to collect topics of national strategic needs and potential breakthroughs in basic research. By focusing on these directions, they aimed to discover new areas of investment and attract talents via the competition. PZL creatively proposed a ‘knockout’ system of the competition, whereby selected experts designed algorithm problems at research frontiers and invited challengers around the world. Successful challengers then in turn designed the next round of algorithm challenges. In addition, a more conventional ‘rating’ system was also included in which well-known problems in AI or big data were chosen for participants worldwide and the best solutions were picked as winners.

## COMPETITION STRUCTURE

With a total award of 10 million RMB, 10 tracks or topics were announced for the ‘knockout’ system and the ‘rating’ system, with 5 for each system. Each track offered a total award of 1 million RMB, with 600 000 RMB for the first prize.

The five topics for the ‘knockout’ system are as follows:

- Chinese Historical Document Analysis and Recognition;
- Robust Adversarial Attack and Defense Algorithms for Deep Learning;
- Tuning Algorithms for Pre-trained Language Models;
- Algorithms for Sample Selection and Label Correction;
- Algorithms for Singular Value Decomposition and Inverse of Nearly Low-rank Matrices.

The five topics for the ‘rating’ system are as follows:

- Character Recognition for Street View Shop Signs;
- Detection Technology for Surface Defect in Industrial Products;
- Algorithms for 3D object detection with Roadside Cameras;
- Object Recognition of Remote Sensing Imagery;
- The Panoptic Scene Graph Generation Challenge.

## ACHIEVEMENTS

In total, 1678 teams with 6634 participants from 439 universities globally and 454 corporations registered for the competition. They mainly came from prestigious universities at home and abroad, including Peking University, Tsinghua University, Sun Yat-sen University, Chinese University of Hong Kong, Ottawa University of Canada and National University of Singapore. In addition, leading tech companies such as Huawei, JD.com, Baidu and Meituan presented their R&D departments as participating teams. The preliminary round selected 150 teams into the finals, from which 86 teams proceeded into the final defense round after individual assessment conducted by third-party organizations. The final defense round was conducted on 28 November 2022 and broadcast live by Guangdong Provincial Station. A total number of 80 teams achieved ranking in the competition and 10 teams won the first prize (algorithm championships).

The launching ceremony and the final defense received widespread media attention (including People's Daily, sina.com, Toutiao, NetEase, stdaily.com, Economy, Science and Education Channel of Guangdong Broadcasting Network). The competition has reached the top level among AI competitions both nationally and internationally in terms of size and the level of competition.

